# High-Quality Genome Sequence of an *Escherichia coli* O157 Strain Carrying an *mcr-1* Resistance Gene Isolated from a Patient in the United States

**DOI:** 10.1128/genomeA.01725-16

**Published:** 2017-03-16

**Authors:** Rebecca L. Lindsey, Dhwani Batra, Lori Rowe, Vladimir N. Loparev, Devon Stripling, Lisley Garcia-Toledo, Kristen Knipe, Phalasy Juieng, Mili Sheth, Haley Martin, Alison Laufer Halpin

**Affiliations:** Centers for Disease Control and Prevention, Atlanta, Georgia, USA

## Abstract

*Enterobacteriaceae* carrying plasmid-mediated colistin resistance have been found around the world. We report here the high-quality whole-genome sequence of an *Escherichia coli* O157:H48 isolate (2016C-3936C1) from Connecticut that carried the *mcr-1* resistance gene on an IncX4-type plasmid.

## GENOME ANNOUNCEMENT

Plasmid-mediated colistin resistance MCR-1 has been identified in *Escherichia coli* and other *Enterobacteriaceae* strains worldwide. IncX4 plasmids carrying the *mcr-1* gene identified in or linked to *E. coli* isolates from human infections have been described in Brazil, China, and South Africa (1–3). We report here the availability of a high-quality whole-genome sequence assembly generated by Illumina and PacBio sequencing and verified using the strain Whole Genome Map (WGM). The sequenced strain was isolated from a Connecticut patient in June 2016 (4) and is Shiga toxin negative.

*Escherichia* genomic DNA was extracted according to the manufacturer’s protocol (Archive Pure; 5 Prime, Gaithersburg, MD). An aliquot of the DNA was used for Illumina MiSeq sequencing, according to the manufacturer’s protocols (Illumina, San Diego, CA). Briefly, DNA was sheared to 600 bp, and libraries were prepared using NEBNext Ultra library prep reagents (New England BioLabs, Ipswich, MA) with dual barcoding indices synthesized in the CDC Biotechnology Core Facility. Sequencing was performed on an Illumina Miseq using a 250 × 250-cycle paired-end sequencing kit. Sequence reads were filtered for read quality, base called, and demultiplexed using Casava (version 1.8.4). Remaining DNA was sheared to 20 kb utilizing needle shearing and used to generate large SMRTbell libraries using the standard library protocols of the Pacific Biosciences DNA template preparation kit (Pacific Biosciences, Menlo Park, CA). The libraries were further size selected utilizing BluePippin (Sage Scientific, Beverly, MA). Cutoff sizes for BluePippin were 10 kb. The finished library was bound to proprietary P6 polymerase and sequenced on a PacBio RSII sequencer using C4 chemistry for 360-min movies. Sequence reads were filtered and assembled *de novo* utilizing the PacBio Hierarchical Genome Assembly Process version 3 (5). WGM were generated according to the OpGen protocol. The sequence order in the resulting PacBio assembly for the chromosome was verified using restriction enzymes NcoI and AflII and WGM.

A single chromosomal contig (Table 1) carried genes for serotype O157:H48, and virulence factors *gad, iha*, *astA*, and *lpfA* were identified. Five plasmids are associated with this isolate (Table 1). pMCR-1-CT carried antimicrobial resistance genes *mcr-1* and an IncX4 origin of replication (http://www.genomicepidemiology.org). These sequences can be found under BioSample accession SAMN06159501, and they were annotated with the NCBI Prokaryotic Genome Automatic Annotation Pipeline (6).

**TABLE 1:** Accession numbers and assembly metrics of *E. coli* 2016C-3936C1 and associated plasmid WGS

Chromosome or plasmid	NCBI accession no.	Genome or plasmid size (bp)	G+C content (%)	Sequence overlap found
2016C-3936C1	CP018770	5,060,618	50.5	Yes
Plasmid 1	CP018771	19,788	52.4	Yes
Plasmid 2	CP018772	55,104	52.3	No
pMCR-1-CT	CP018773	33,305	41.8	Yes
Plasmid 4	CP018774	95,419	50.0	No
Plasmid 5	CP018775	174,846	49.6	No

A detailed report on additional analyses of the draft genome and plasmid sequences will be included in a future publication.

### Accession number(s).

The annotated whole-genome *E. coli* O157:H48 sequences have been deposited in DDBJ/ENA/GenBank under the accession numbers listed in Table 1. The version described in this paper is the first version.
